# The Role of Aerobic Training Variables Progression on Glycemic Control of Patients with Type 2 Diabetes: a Systematic Review with Meta-analysis

**DOI:** 10.1186/s40798-019-0194-z

**Published:** 2019-06-07

**Authors:** Rodrigo Sudatti Delevatti, Cláudia Gomes Bracht, Salime Donida Chedid Lisboa, Rochelle Rocha Costa, Elisa Corrêa Marson, Nathalie Netto, Luiz Fernando Martins Kruel

**Affiliations:** 1 Universidade Federal de Santa Catarina, Office 215, Deputado Edu Antônio Vieira St., Administrative Center, Sports Center, Pantanal District, Florianópolis, 88036-120 Brazil; 2 Universidade Federal do Rio Grande do Sul, Porto Alegre, Brazil

**Keywords:** Exercise, Glycated hemoglobin, Diabetes mellitus

## Abstract

**Background:**

Aerobic training (AT) improves glycemic control in patients with type 2 diabetes. However, the role of the progression of training variables remains unclear. The objective of this review was to analyze the effects of progressive AT (PAT) and non-progressive AT (NPAT) on glycated hemoglobin (HbA1c) in patients with type 2 diabetes.

**Methods:**

Data sources used were PubMed, Cochrane Central, Embase, SPORTDiscus, and LILACS. Studies that evaluated the effect of at least 12 weeks of PAT and NPAT compared to a control condition on HbA1c levels in type 2 diabetes patients were eligible for analysis. Two independent reviewers screened the search results, extracted the data, and assessed the risk of bias. Effect sizes (ESs) were calculated using the standardized mean difference in HbA1c levels between the intervention and control groups using a random-effect model.

**Results:**

Of 5848 articles retrieved, 24 randomized clinical trials (825 participants) were included. Among the included studies, 92% reported to have performed a randomization process, 8% presented allocation concealment, 21% reported blinding of outcome assessment, and 38% reported complete outcome data. AT reduced HbA1c levels by 0.65% (ES: − 1.037; 95% confidence interval [CI]: − 1.386, − 0.688; *p* < 0.001). The reduction in HbA1c induced by PAT was 0.84% (ES: − 1.478; 95% CI − 2.197, − 0.759; *p* < 0.001), and NPAT was 0.45% (ES: − 0.920; 95% CI − 1.329, − 0.512; *p* < 0.001). Subgroup analysis of the different forms of progression showed a reduction in HbA1c levels of 0.94% (ES: − 1.967; 95% CI − 3.783, − 0.151; *p* = 0.034) with progression in volume, 0.41% (ES: − 1.277; 95% CI − 2.499, − 0.056; *p* = 0.040) with progression in intensity, and 1.27% (ES: − 1.422; 95% CI − 2.544, − 0.300; *p* = 0.013) with progression in both volume and intensity. Subgroup analysis of the different modalities of AT showed a reduction of 0.69% (ES: − 1.078; 95% CI − 1.817, − 0.340; *p* = 0.004) with walking and/or running and of 1.12% (ES: − 2.614; 95% CI − 4.206, − 1.022; *p* = 0.001) with mixed protocols while progressive training was adopted. In non-progressive protocols, a significant HbA1c reduction was only found with walking and/or running (− 0.43%; ES: − 1.292; 95% CI − 1.856, − 0.72; *p* < 0.001).

**Conclusion:**

The effect of PAT on glycemic control was greater than that of NPAT, especially when volume and intensity were progressively incremented throughout the interventions.

**Electronic supplementary material:**

The online version of this article (10.1186/s40798-019-0194-z) contains supplementary material, which is available to authorized users.

## Key Points

- Progressive aerobic training provides HbA1c reduction of greater magnitude than non-progressive aerobic training.
- Among progression strategies in aerobic training, greater reductions in HbA1c levels occur with progression in duration and intensity, followed by duration progression, with lower reduction by intensity progression.
- Reductions in HbA1c levels of higher magnitude occur in patients with type 2 diabetes without comorbidities, untrained, that performing walking and/or running or a mix of modalities.

## Background

Physical training is an important non-pharmacological intervention for type 2 diabetes management [1–5]. Among the available forms of training, aerobic exercise training (AT) is supported by strong evidence for its benefits on outcomes such as blood pressure [6], systemic inflammation [7], cardiorespiratory fitness [8], and glycemic control, evaluated especially by glycated hemoglobin (HbA1c) [3, 4, 8], which is considered primary outcome in the diabetes treatment [3, 9].

Aside from the benefits to these different outcomes, many studies have investigated the role of different training variables, such as duration [9, 10], intensity [11–13], weekly frequency [14], and characteristics such as training supervision [10, 15] and training environment (aquatic or dry-land) [16–19], on type 2 diabetes control, with a particular focus on HbA1c reduction. For this goal, the current recommendations for structured AT include training preferably supervised, with weekly duration of at least 150 min of moderate to vigorous intensity performed in three or more sessions per week and with no more than 2 days between exercise sessions. Endurance training of greater intensity and shorter duration (≥ 75 min/week) has also been recommended for younger and more physically fit patients [3, 4]. However, despite these recommendations, well-conducted clinical trials [20–22] in which the AT was prescribed according to these recommendations but with minimal or no progression in physiological/internal load no found HbA1c reductions. Meanwhile, some interventional studies that did not follow these recommendations but progressed training in terms of volume and/or intensity throughout the intervention found HbA1c reductions [12, 16, 23, 24]. These findings show that the optimization of glycemic control by AT may not only depend on training dosage (volume and intensity) but also on the progression of the volume and/or intensity of training.

Current perspectives [3, 4] on exercise for the type 2 diabetes management indicate that over time, the intensity, frequency, and/or duration of training should be increased. Nevertheless, there is a lack of consistent evidence, including well-conducted randomized controlled trials (RCTs) and systematic reviews with meta-analyses, evaluating the progression of training variables and comparing effects of AT with and without progression (PAT and NPAT) on health outcomes, especially on HbA1c. Additionally, in the context of exercise intervention for type 2 diabetes control in clinical practice, there is little evidence for the effects of different progressive training strategies that would allow the magnitude of reduction in HbA1c levels caused by the progression of training volume (frequency and/or duration), intensity, or both (volume and intensity), issues to be clarified. Advancing the understanding of this specific question will make it possible to analyze the advantages and disadvantages of the different AT progression strategies and of non-progressed AT, which is a normal exercise prescription for the people with type 2 diabetes. Thus, the aim of this study was to perform a systematic review and meta-analysis of studies analyzing the effects of AT with and without progression on HbA1c in patients with type 2 diabetes.

## Methods

This study was a systematic review and meta-analysis of RCTs performed according to the Cochrane Handbook for Systematic Reviews of Interventions [25]. Results are reported according to Preferred Reporting Items for Systematic Reviews and Meta-Analyses (PRISMA) [26].

### Data Sources and Searches

PubMed, Cochrane Central, Embase, SPORTDiscus, and LILACS databases were searched for eligible publications in October 2015 and updated in September 2017. In addition, a manual search was conducted of the reference lists of located studies. When the same result was found in different studies, only one (the first to be published) was included. Searches were made without year limitation. The following search terms were used in combination and/or alone: “Diabetes Mellitus, type 2,” “Exercise,” and “Randomized controlled trial.” The Boolean operators “OR” and “AND” were used to search the databases. The searches were conducted using MeSH terms and their respective synonyms. All databases were primarily searched using the same keywords. The search of the PubMed database is shown in Additional file 1.

### Eligibility Criteria

RCTs published in English, Portuguese, and Spanish that included adults (≥ 18 years) of both sexes with type 2 diabetes who were exposed to at least 12 weeks of structured and supervised AT were included. We considered AT as all training protocol in which the exercises movements had endurance features, without resistance or stretching features, mobilizing large muscle groups, usually in cyclic activities performed by lower limbs. There were no restrictions concerning the modality, intensity, session duration, volume, and weekly frequency of AT. Clinical trials that included comparisons between at least one group performing AT and a control group without exercise intervention were included. In the studies in which there was an AT and nutritional counseling group and a group treated with nutritional counseling only, the latter was considered as the control group as this controls for physical exercise better than a group not exposed to any intervention. To be eligible, the studies had to provide the pre- and post-intervention values of HbA1c or differences between means with their respective dispersion values. All the studies in which AT was associated with another type of physical exercise were excluded, as were studies that did not clearly describe the frequency, duration, and intensity of AT. When intervention characteristics could not be understood, the respective authors were contacted via email. Studies that performed any change(s) in the intensity and/or volume variables, such as session duration and/or frequency during the period of intervention, were considered to involve PAT, whereas studies that maintained the intensity and volume of the exercises at fixed levels during the entire period of intervention were considered to involve NPAT.

### Study Selection and Data Extraction

The titles and abstracts of retrieved articles were independently assessed by two investigators (C.G.B and E.C.M), who independently read all titles and abstracts. Then, the reviewers independently evaluated the full-text articles and determined study eligibility by examining the articles. After this independent selection, the investigators compared the studies to determine if there was any discordance. If there was, it was resolved by consensus or, if needed, in consultation with a third reviewer (R.S.D).

Data extraction was independently performed by the same investigators and the results were compared to avoid mistakes in the extraction process. If there were any discrepancies, they were discussed between the investigators and solved by consensus with a third investigator (R.S.D) if needed. For all studies, data extraction was conducted with a standardized form to collect the following data: author and year, participants, intervention, and outcomes. With respect to participants, mean age, training status, comorbidities, glucose lowering drugs users, diagnosis duration, and nutritional co-intervention were extracted. With respect to interventions, data concerning training modality, intervention period, session duration, weekly frequency, intensity, and volume were extracted. As the outcome, the HbA1c values (mean and standard deviations or change values with their respective dispersion measures) of the AT groups and control groups before and after the training period were extracted.

### Assessment of Risk of Bias

Risk of bias was independently assessed by two investigators (C.G.B and E.C.M). If there was any discordance, it was solved by consensus or, if needed, in consultation with a third reviewer (R.S.D). The assessment was performed by considering the following criteria: random sequence generation, allocation concealment, blinding of outcome assessment, and incomplete outcome data. Only blinding of assessors was considered due to the impossibility of blinding the participants and personnel in studies involving exercise. Studies without a clear description of random sequence generation or information about how the allocation list was blinded were considered as not having fulfilled these criteria. The risk of bias was evaluated in the following form: high risk (when the methodological criteria, such as adequate sequence generation, were not reported or were not performed); low risk (when the methodological criteria were performed appropriately); unclear risk (when there was no adequate description of the criteria, making it difficult to evaluate the risk as high or low). Risk of bias in RCTs was evaluated according to the Cochrane Handbook [25].

### Data Analysis

The pooled effect estimates were computed from the change scores between the baseline and the end of intervention, their standard deviations, and the number of participants. Data from intention-to-treat analysis were entered whenever available in the included studies.

Results are presented as the standardized mean differences, and calculations were performed using random effects models. Statistical heterogeneity of treatment effects among studies was evaluated by Cochran’s *Q* test and the *I*^2^ inconsistency test. It was considered that values > 50% indicated high heterogeneity [25]. Subgroup analyses were conducted for training progression (no progression, any type of progression, intensity progression, volume progression, and volume and intensity progression), training status of participants, presence of comorbidities, and AT modality. Meta-regression analyses were performed to investigate potential confounders: mean age (years), body mass index (BMI) (kg/m^2^), follow-up duration (weeks), body mass (kg), weekly frequency (number of sessions per week), percentage of women in the sample (%), diagnosis duration (years), baseline HbA1c, number of users of glucose-lowering medications, session duration (min), and weekly duration (min).

Furthermore, publication bias was assessed using funnel plots for each outcome (of each trial’s effect size against the standard error). Funnel plot asymmetry was evaluated using Begg’s and Egger’s tests [27] and significant publication bias was considered when the *p* value was < 0.05. The trim-and-fill computation was used to estimate the effect of publication bias on the interpretation of the results.

Forest plots were generated to present the pooled effect and the standardized mean differences with 95% confidence intervals (CIs). Statistical significance was set at a *p* value < 0.05. All analyses were performed using Comprehensive Meta-Analysis Software version 3.3.070.

## Results

### Study Selection

**Fig. 1:**
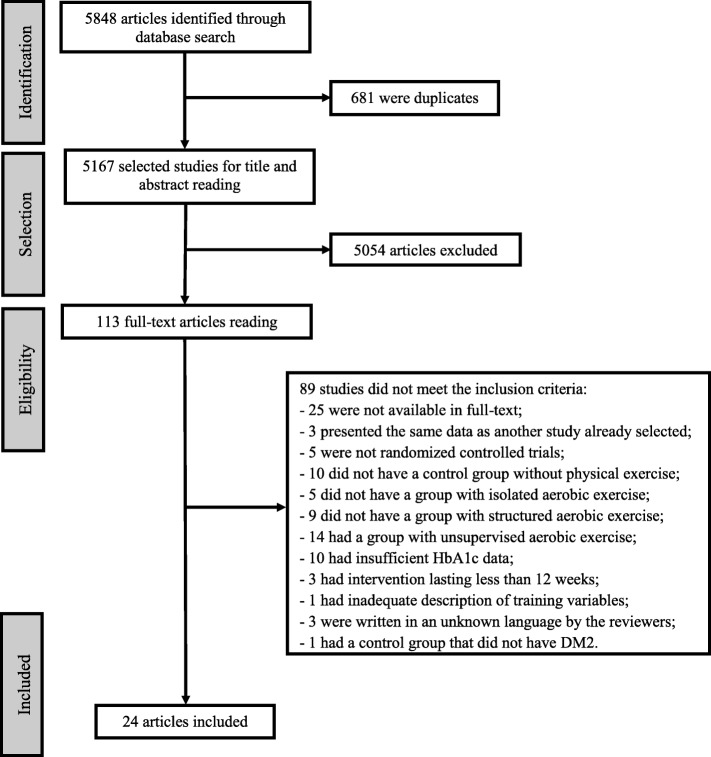
Flow of information through the different phases of the systematic review

The data base search yielded a total of 5848 studies. After adjusting for duplicates, 5167 studies remained. Of these, 5054 were discarded because they did not meet the eligibility criteria. Thus, the full texts of the remaining 113 studies were examined in more detail. Of these, 89 did not meet the inclusion criteria. Thus, 24 studies [12, 20–23, 28–46] met the inclusion criteria and were included in the quantitative analysis (Fig. 1). Of these, three trials [21, 23, 45] were included twice because they met the eligibility criteria for two comparison groups. No additional studies were identified by checking the references of the included studies and no relevant unpublished studies were obtained.

Of the 24 studies included, 12 involved progression of training variables [12, 23, 28–35, 44, 45]. Of these, two studies were comprised of two intervention groups, resulting in a total of 14 compared groups. The remaining 12 studies did not involve progression [20–22, 36–43, 46]. One of these studies included two intervention groups, resulting in 13 compared groups.

### Characteristics of Studies

In total, 825 participants were included in the meta-analysis. Among these, 489 and 336 participants were included in the AT and control groups, respectively. The majority of the studies (54.2%) analyzed both sexes, whereas 20.8% analyzed only women, 8.3% analyzed only men, and 16.6% did not report the sex of the participants. Most studies included previously untrained individuals (66.6%) and 33.4% did not make the training status of the participants clear. Table 1 displays the characteristics of the 24 included studies.

**Table 1:** Characteristics of the included studies

Study	Age (years) Mean ± SD	HbA1c baseline (%) or change (%) Mean ± SD	Training status	Comorbidities	Nutritional co-intervention	Adverse events	Adherence (%)	Drop-outs
Progressive aerobic training								
Alvarez et al. 2016 [12]	45 ± 2	I: 7.0 ± 0.5 C: 7.4 ± 0.5	Untrained	Overweight and obesity	No	Post-exercise hypoglycemia	89	I: 0% C: 29%
Belli et al. 2011 [28]	54 ± 2	I: 6.8 ± 0.4 C: 7.2 ± 0.5	Untrained	NR	No	NR	92	I: 33% C: 20%
Kadoglou et al. 2007 [35]	61 ± 4	I: − 0.6 ± 0.4* C: 0.3 ± 0.1*	Untrained	NR	No	No	NR	I: 3% C: 11%
Kadoglou et al. 2012 [44]	58 ± 6	I: − 0.6 ± 0.1* C: − 0.05 ± 0.01*	Untrained	Overweight and obesity	Standard nutritional prescription, orientation	No	NR	I: 10% C: 14%
Lambers et al. 2008 [29]	54 ± 8	I: 7.4 ± 1.7 C: 6.7 ± 0.9	NR	Obesity, cardiovascular risk	No	Hypoglycemia	85%	I: 28% C: 17%
Mitranun et al. 2014 [23]	61 ± 2	I (Interval): 7.6 ± 0.3 I (Continuous): 7.7 ± 2.0 C: 7.8 ± 2.0	Untrained	NR	No	NR	At least 80%	I: 7% C: 13%
Negri et al. 2010 [30]	65 ± 5	I: 7.5 ± 0.2 C: 7.4 ± 0.5	Untrained	NR	No	Hypoglycemia	At least 50%	I: 33% C: 5%
Oliveira et al. 2012 [31]	52 ± 9	I: 7.4 ± 1.8 C: 7.0 ± 0.7	Untrained	NR	No	Hypoglycemia, hypotension	NR	I: 11% C: 31%
Sentinelli et al. 2014 [32]	57 ± 7	I: 7.1 ± 1.3 C: 7.1 ± 1.3	Untrained	NR	No	NR	NR	I: 0% C: 0%
Tomar et al. 2013 [33]	43 ± 10	I: 8.3 ± 3.5 C: 8.9 ± 5.9	Untrained	NR	No	No	100%	I: 17% C: 0%
Vancea et al. 2009 [45]	57 ± 6	I: − 0.8 ± 1.4* C: − 0.3 ± 0.4*	NR	NR	No	NR	NR	NR
Yavari et al. 2012 [34]	49 ± 8	I: 8.5 ± 1.1 C: 8.7 ± 1.1	Untrained	NR	No	NR	80%	I: 19% C: 19%
Non-progressive aerobic training								
Blonk et al. 1994 [36]	58.7*	I: − 1.0 ± 4.7* C: − 0.1 ± 9.5*	NR	NR	Yes	NR	NR	I: 12% C: 15%
Church et al. 2010 [20]	56 ± 8	I: 7.5 ± 0.6 C: 7.61 ± 0.64	Untrained	NR	No	No	NR	NR
Emereziani et al. 2015 [37]	66 ± 4	I: 6.7 ± 1.6 C: 7.0 ± 1.5	Untrained	NR	Yes	NR	NR	NR
Giannopoulou et al. 2005 [38]	57 ± 2	I: 6.8 ± 0.5 C: 7.3 ± 0.5	Untrained	Obesity	Yes	NR	NR	I: 21% C: 39%
Jorge et al. 2011 [22]	53 ± 9	I: 7.6 ± 1.7 C: 6.9 ± 0.7	Untrained	Overweight and obesity	No	NR	97%	I: 10% C:31%
Karstoft et al. 2013 [21]	58 ± 2	I (Continuous): 6.6 ± 0.2 I (Interval): 6.9 ± 0.2 Control: 6.4 ± 0.2	Untrained	NR	No	NR	89%	I: 9% C: 0%
Kwon et al. 2011 [39]	65 ± 6	I: 7.5 ± 0.7 C: 7.2 ± 0.8	Untrained	Overweight	No	NR	NR	I: 9% C: 0%
Midlebrooke et al. 2006 [40]	63 ± 7	I: 6.8 ± 0.9 C: 7.2 ± 1.1	Physically active and untrained	Proliferative retinopathy, sensory neuropathy, autonomic neuropathy, microalbuminuria	No	No	80%	I: 47% C: 0%
Nuttamonwarakul et al. 2012 [46]	> 60	I: 7.7 ± 1.1 C: 7.6 ± 0.2	NR	NR	No	NR	NR	NR
Parra-Sanchez et al. 2015 [41]	72 ± 4	I: 7.0 ± 0.9 C: 7.3 ± 1.1	Untrained	NR	No	No	NR	I: 6% C: 22%
Shenoy et al. 2009 [42]	55 ± 5	I: 8.1 ± 0.9 C: 7.8 ± 0.9	Untrained	NR	No	No	NR	NR
Yan et al. 2014 [43]	54 ± 2	I: 8.8 ± 0.5 C: 8.4 ± 0.9	Physically active	NR	No	NR	NR	NR

*I* intervention, *C* control, *HbA1c* glycated hemoglobin. * indicates values of HbA1c demonstrated in changes (post-pre intervention and control) and not by baseline values

### Characteristics of Interventions

#### Overview Findings (All Interventions)

In relation to the AT protocols, walking was the most often reported modality, followed by cycle ergometry and then the combination of walking, running, and cycle ergometry. Concerning the methods of AT used in 27 groups of exercise, there were only three groups (11%) that used interval training, whereas the other 24 groups (89%) trained in a continuous mode. Even those studies that did not clearly report the methods used but described only one intensity and one duration per session were considered as implementing a continuous training method. The intervention periods ranged from 12 to 52 weeks, with sessions lasting 20–90 min and 2–5 sessions performed per week.

#### Progressive Aerobic Training

The mean of the intervention periods was of 19 ± 11 weeks (ranging from 12 to 52 weeks). Mean session duration was 32 ± 14 min (from 20 to 60 min) in the beginning of interventions and 46 ± 17 min (from 30 to 90 min) in the end of interventions. Two studies had four weekly sessions, where as one study had five weekly session and the others 11 interventions had three weekly sessions. Regarding to mean weekly duration, interventions had 105 ± 49 min (from 60 to 180 min) in the beginning of interventions and 151 ± 50 min (from 90 to 270 min) in the end of interventions. In relation to intensity, only two studies used heart rate reserve (HR_res_) percentages as prescription method, being one study with HIIT, in which stimuli were always from 90 to 100% and other study with progression in intensity, ranging from 60 to 85%_._ Other two studies used metabolic thresholds for prescription (ventilatory and lactate thresholds were used). Maximum oxygen uptake (VO_2max_) percentages were used in three studies, with mean intensity of 50 ± 0% and 75 ± 10% (from 65 to 85%) in the beginning and end of interventions, respectively. Percentages of maximum heart rate (HR_max_) was the most used method (five protocols), with mean intensity of 57 ± 7% (from 40 to 60%) and 73 ± 3% (from 70 to 75%) for the beginning and end of interventions, respectively. Another two studies described the intensity trained only as low, moderate, and high, without a specific method of prescription and control.

Finally, analyzing all the training variables in the end of the interventions separately, ten interventions of PAT are in agreement with the AT recommendations for diabetes control [3].

#### Non-Progressive Aerobic Training

The mean of the intervention periods was 18 ± 9 weeks (ranging from 12 to 39 weeks). Mean session duration was 44 ± 13 min (from 30 to 60 min). Three studies had only two weekly sessions, three studies had five weekly sessions, whereas other study had the possibility of reaching the determined volume in 3–5 weekly sessions and the others seven interventions had three weekly sessions. Regarding mean weekly duration, interventions had 155 ± 88 min (from 60 to 300 min). In relation to intensity, three studies prescribed it by HR_max_ percentages, with mean intensity of 72 ± 3% (from 60 to 80%). Six interventions used VO_2max_ or VO_2peak_ percentages, with mean intensity of 64 ± 9% (from 50 to 80%). Two studies used metabolic thresholds for prescription (aerobic threshold and lactate thresholds were used). One study reported the intensity based on talking capacity and another study described the intensity trained only as moderate, without a method of prescription and control.

As with PAT, by analyzing the studies separately, NPAT showed ten interventions that are in agreement with AT recommendations for diabetes control [3]. The complete characteristics of the interventions are presented in Table 2.

**Table 2:** Characteristics of the interventions

Study	Modality (*n*)	Intervention period	Session duration	Weekly frequency	Weekly duration	Intensity	Volume
Progressive aerobic training							
Alvarez et al. 2016 [12]	Interval: stimulus—running, recovery—walking (*n* = 13) Control (*n* = 10)	16 weeks	Beginning: 22 min Final: 37.5 min	3	Beginning: 66 min Final: 112.6 min	Stimulus: 90–100% HR_res_ Recovery: ≤ 70% HR_res_	–
Belli et al. 2011 [28]	Walking (*n* = 9) Control (*n* = 10)	12 weeks	Beginning: 20 min Final: 60 min	3	Beginning: 60 min Final: 180 min	HR_VT_	–
Kadoglou et al. 2007 [35]	Walking or running on the treadmill/cycle ergometer (*n* = 29) Control (*n* = 27)	26 weeks	Beginning: 30 min Final: 45 min	4	Beginning: 120 min Final: 180 min	Beginning: 50%VO_2peak_ Gradual increase throughout the 26 weeks Final: 75%VO_2peak_	–
Kadoglou et al. 2012 [44]	Walking or running on the treadmill/cycle ergometer (*n* = 21) Control (*n* = 7)	26 weeks	Gradual increases until 4th week, reaching 45 min	4	180 min	Beginning: 60% HR_max_ Final: 75% HR_max_	–
Lambers et al. 2008 [29]	Walking or running/cycle ergometer (*n* = 18) Control (*n* = 6)	12 weeks	50 min	3	150 min	Beginning: 60%HR_res_ Middle: 75%HR_re_ Final: 85%HR_re_	–
Mitranun et al. 2014 [23]	Interval training on the treadmill (it does not report if it is walking or running in the stimulus and recovery) (*n* = 14) Control (*n* = 8)	12 weeks	Beginning: 20 min Middle: 20 min Final: 30 min	3	Beginning: 60 min Middle: 60 min Final: 90 min	Beginning: 50% VO_2peak_ Middle: 80% VO_2peak_ Final: 85% VO_2peak_	Beginning: 33.6 L O_2_ Middle: 36.2 L O_2_ Final: 53.7 L O_2_
	Continuous on the treadmill (does not report if it is walking or running) (*n* = 14) Control (*n* = 8)		Beginning: 20 min Middle: 20 min Final: 30 min	3	Beginning: 60 min Middle: 60 min Final: 90 min	Beginning: 50% VO_2peak_ Middle: 60% VO_2peak_ Final: 65% VO_2peak_	Beginning: 33.6 L O_2_ Middle: 36.2 L O_2_Final: 53.7 L O_2_
Negri et al. 2010 [30]	Walking (*n* = 21) Control (*n* = 20)	16 weeks	45 min	3	135 min	Beginning: low(NS) Final: moderate (NS)	–
Oliveira et al. 2012 [31]	Cycle ergometer (*n* = 11) Control (*n* = 4)	12 weeks	Beginning: 20 min Final: 50 min	3	From 60 to 150 min	HR_LT_	–
Sentinelli et al. 2014 [32]	Nordic walking (*n* = 10) Control (*n* = 10)	12 weeks	Beginning: 60 min Final: 90 min	3	Beginning: 180 min Final: 270 min	Beginning: low to moderate (NS) Final: moderate to high (NS)	Beginning: 4–5 km with slope of 7% of inclination Final: 7 km with slope of 14% of inclination
Tomar et al. 2013 [33]	Walking or running on the treadmill/cycle ergometer (*n* = 12) Control (*n* = 12)	12 weeks	NS	3	Increased every 4 weeks (NS)	Beginning: 40–50% HR_max_; Gradual increase (NS)	–
Vancea et al. 2009 [45]	Walking (*n* = 14) Control (*n* = 10)	20 weeks	30 min	3	90 min	Beginning: 60% HR_max_ Final: 70% HR_max_	–
	Walking (*n* = 9) Control (*n* = 7)		30 min	5	150 min	Beginning: 60% HR_max_ Final: 70% HR_max_	–
Yavari et al. 2012 [34]	Treadmill/elliptical/cycle ergometer (*n* = 20) Control (*n* = 7)	52 weeks	Beginning: 20 min Final: 60 min	3	Beginning: 60 min Final: 180 min	Beginning: 60% HR_max_ Final: 75% HR_max_	–
Non-progressive aerobic training							
Blonk et al. 1994 [36]	Cycle ergometer (*n* = 26) Control (*n* = 27)	26 weeks	30 min	2	60 min	60–80%HR_max_	–
Church et al. 2010 [20]	Walking (*n* = 52) Control (*n* = 16)	39 weeks	50 min	3	150 min	50–80% VO_2max_	12 kcal/kg/week
Emereziani et al. 2015 [37]	Walking/cycle ergometer (*n* = 15) Control (*n* = 15)	12 weeks	30 min	2	60 min	HR_AT_	–
Giannopolou et al. 2005 [38]	Walking (*n* = 11) Control (*n* = 6)	14 weeks	50 min	3	150 min	65–70% VO_2peak_	Deficit of 200 kcal
Jorge et al. 2011 [22]	Cycle ergometer (*n* = 12) Control (*n* = 4)	12 weeks	60 min	3	180 min	HR_LT_	–
Karstoft et al. 2013 [21]	Walking (continuous) (*n* = 11) Control (*n* = 4)	17 weeks	60 min	5	300 min	55% VO_2peak_	–
	Walking (interval) (*n* = 11) Control (4)		60 min	5	300 min	70% VO_2peak_ (3 min above, 3 min below)	–
Kwon et al. 2001 [39]	Walking (*n* = 13) Control (*n* = 8)	12 weeks	60 min	5	300 min	Moderate (NS)	4 to 6 METs
Middlebrooke et al. 2006 [40]	Not reported (*n* = 15) Control (*n* = 30)	26 weeks	30 min	3	90 min	70–80% HR_max_	–
Nuttamon- warakul et al. 2012 [46]	Aquatic training (*n* = 20) Control (*n* = 20)	12 weeks	30	3	90 min	70% HR_max_	–
Parra-Sanchez et al. 2015 [41]	Walking (*n* = 47) Control (*n* = 41)	12 weeks	40 min	2	80 min	Velocity which allowed to speak without stuttering	–
Shenoy et al. 2009 [42]	Walking (*n* = 10) Control (*n* = 5)	16 weeks	30 min	3	90 min	–	–
Yan et al. 2014 [43]	Not reported (low intensity) (*n* = 22) Control (*n* = 6)	12 weeks	45 min	3–5	135 to 225 min	50% VO_2max_	–
	Not reported (high intensity) (*n* = 9) Control (*n* = 4)		45 min	3	135 min	75% VO_2max_	–

*Min* minutes, *n* number of participants, *HR*_*res*_ heart rate reserve, *HR*_*max*_ maximum heart rate, *HR*_*LT*_ heart rate corresponding to lactate threshold, *HR*_*AT*_ heart rate corresponding to aerobic threshold, *HR*_*VT*_ heart rate corresponding to ventilatory threshold, *VO*_*2peak*_ peak oxygen consumption, *L O*_2_ liters of oxygen, *NS* not specified. The study of Blonk et al. (1994) did not state the SD of the participants’ age

#### Analysis of the Risk of Bias

Among the included studies, 92% (22 of 24) reported to have performed a randomization process for the allocation of the participants into the study groups, 8% (two of 24) presented allocation concealment, 21% (five of 24) reported blinding of outcome assessment, and 62% (15 of 24) reported incomplete outcome data (Table 3).

**Table 3:** Risk of bias

Study	Random sequence generation	Allocation concealment	Blinding of outcome assessment	Incomplete outcome data
Alvarez et al. 2016 [12]	Low	High	Low	High
Belli et al. 2011 [28]	Low	High	High	High
Kadoglou et al. 2007 [35]	Low	High	High	High
Kadoglou et al. 2012 [44]	Low	High	High	High
Lambers et al. 2008 [29]	Low	Low	Low	High
Mitranun et al. 2014 [23]	Low	High	High	High
Negri et al. 2010 [30]	Low	High	High	High
Oliveira et al. 2012 [31]	Low	High	High	High
Sentinelli et al. 2014 [32]	Low	High	High	Low
Tomar et al. 2013 [33]	Low	High	High	Low
Vancea et al. 2009 [45]	Low	High	High	Low
Yavari et al. 2012 [34]	Low	High	High	Low
Blonk et al. 1994 [36]	High	High	Unclear	High
Church et al. 2010 [20]	Low	High	Low	Low
Emereziani et al. 2015 [37]	Low	High	High	Low
Giannopoulou et al. 2005 [38]	High	High	High	High
Jorge et al. 2011 [22]	Low	High	High	Low
Karstoft et al. 2013 [21]	Low	High	Low	High
Kwon et al. 2011 [39]	Low	High	High	High
Midlebrooke et al. 2006 [40]	Low	High	High	High
Nuttamonwarakul et al. 2012 [46]	Low	High	High	Low
Parra-Sanchez et al. 2015 [41]	Low	Low	Low	High
Shenoy et al. 2009 [42]	Low	High	High	High
Yan et al. 2014 [43]	Low	High	High	Low

### Effects of Interventions

#### Effectiveness of AT (PAT and NPAT)

In general, AT was associated with a reduction in HbA1c of 0.65% compared with no intervention (effect size: − 1.037; 95% CI − 1.386, − 0.688; *p* < 0.001; *I*^2^: 76%). The analysis of publication bias for this outcome showed no significant bias (*p* = 0.139).

### Effectiveness of Progressive Aerobic Training

#### Meta-analysis Results (Progressive)

**Fig. 2:**
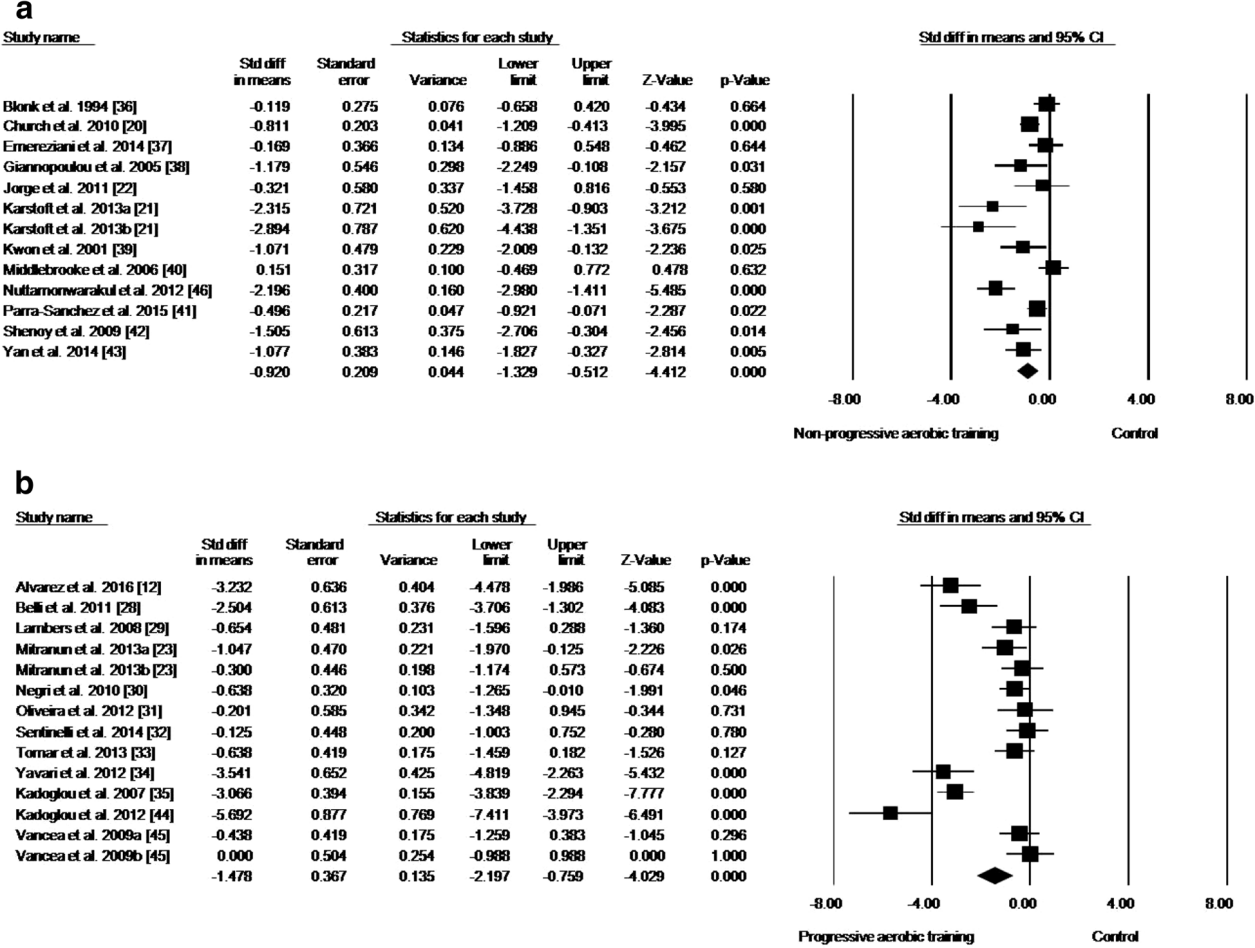
**a** Standard mean differences in HbA1c observed with non-progressive aerobic training and control (no intervention). **b** Standard mean differences in HbA1c with progressive aerobic training and control (no intervention). (Black square) Study-specific estimates; (black diamond) pooled estimates of random-effects meta-analyses. *Std diff* standardized difference, *CI* confidence interval. Letters (a and b) subscribed indicate different aerobic training protocols in a same study

Data concerning HbA1c reductions in response to PAT were available from 12 studies, which compared PAT versus control groups in a total of 353 participants (PAT groups = *n*: 215; control groups = *n*: 138) (Fig. 2; Table 4). PAT was associated with a reduction in HbA1c of 0.84% compared with no intervention (effect size: − 1.478; 95% CI − 2.197, − 0.759; *p* < 0.001; *I*^2^: 87%). The analysis of publication bias showed significant bias (*p* = 0.049), but after adjustment (according to Duval and Tweedie’s trim and fill test), the effect size remained the same identified by the meta-analysis (− 1.478).

**Table 4:** Meta-analysis results

Analysis and sub-analysis	Number of comparisons	Meta-analysis				Heterogeneity	
		Difference in means (%)	Effect size	95% CI	*p* value	*I* ^2^	*p* value
Aerobic training	24	− 0.65	− 1.037	− 1.386; − 0.688	< 0.001	76%	< 0.001
Non-progressive aerobic training	13	− 0.45	− 0.920	− 1.329; − 0.512	< 0.001	74%	< 0.001
Presence of comorbidities	6	− 0.33	− 0.554	− 1.048; − 0.061	0.028	52%	0.065
Absence of comorbidities	3	− 0.79	− 1.358	− 2.556; − 0.159	0.026	86%	< 0.001
Walking/running modalities	7	− 0.43	− 1.292	− 1.856; − 0.727	< 0.001	61%	0.018
Cycle ergometer modalities	2	–	− 0.156	− 0.643; 0.330	0.529	0%	0.753
Untrained participants	9	− 0.39	− 0.956	− 1.382; − 0.530	< 0.001	58%	0.013
Undefined participant’s training status	2	–	− 0.443	− 1.646; 0.759	0.470	84%	0.013
Progressive aerobic training	14	− 0.84	− 1.478	− 2.197; − 0.759	< 0.001	87%	< 0.001
Presence of comorbidities	4	− 0.79	− 3.061	− 4.823; − 1.299	0.001	90%	< 0.001
Absence of comorbidities	9	− 0.94	− 0.699	− 1.226; − 0.171	0.009	68%	0.001
Walking/running modalities	7	− 0.69	− 1.078	− 1.817; − 0.340	0.004	78%	< 0.001
Mixed modalities	5	− 1.12	− 2.614	− 4.206; − 1.022	0.001	92%	< 0.001
Untrained participants	11	− 0.99	− 1.808	− 2.688; − 0.927	< 0.001	89%	< 0.001
Undefined participant’s training status	3	–	− 0.381	− 0.905; 0.143	0.154	0%	0.633
Progressive aerobic training (intensity)	6	− 0.57	− 1.625	− 2.903; − 0.348	0.013	92%	< 0.001
Progressive aerobic training (duration)	4	− 0.93	− 2.264	− 3.603; − 0.926	< 0.001	84%	< 0.001
Progressive aerobic training (intensity and duration)	6	− 1.27	− 1.422	− 2.544; − 0.300	0.013	89%	< 0.001

#### Subgroup Analysis (Progressive)

To investigate the possible causes of the high heterogeneity found in this analysis (*p* < 0.001), we conducted some subgroup analyses. These analyses suggested that the effectiveness of PAT for reducing HbA1c was not influenced by the presence of comorbidities. Trials that included participants with comorbidities were associated with significant HbA1c reductions of 0.79% (effect size: − 3.061; 95% CI − 4.823, − 1.299; *p* = 0.001; *I*^2^: 90%), as were those that did not include participants with comorbidities, which reported a decrease of 0.94% (effect size: − 0.699; 95% CI − 1.226, − 0.171; *p* = 0.009; *I*^2^: 68%). When analyzed according to the specific modality of AT, an HbA1c reduction of 0.69% (effect size: − 1.078; 95% CI − 1.817, − 0.340; *p* = 0.004; *I*^2^: 78%) for walking and/or running and 1.12% (effect size: − 2.614; 95% CI − 4.206, − 1.022; *p* = 0.001; *I*^2^: 92%) for mixed protocols (combination of different ergometers) was detected.

When divided according to training status, the studies involving untrained patients showed an HbA1c reduction of 0.99% (effect size: − 1.808; 95% CI − 2.688, − 0.927; *p* < 0.001; *I*^2^: 89%), whereas studies lacking a clear description of the participants training status did not detect an HbA1c reduction (effect size: − 0.381; 95% CI − 0.905, 0.143; *p* = 0.154; *I*^2^: 0%).

**Fig. 3:**
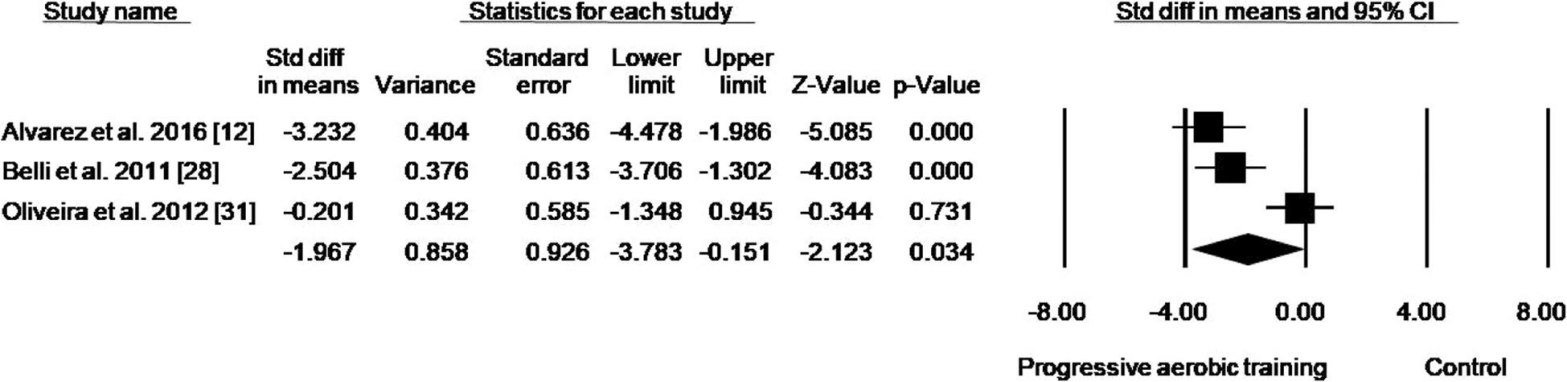
Standard mean differences in HbA1c observed with aerobic training progressing in duration and control (no intervention). (Black square) Study-specific estimates; (black diamond) pooled estimates of random-effects meta-analyses. *Std diff* standardized difference, *CI* confidence interval

**Fig. 4:**
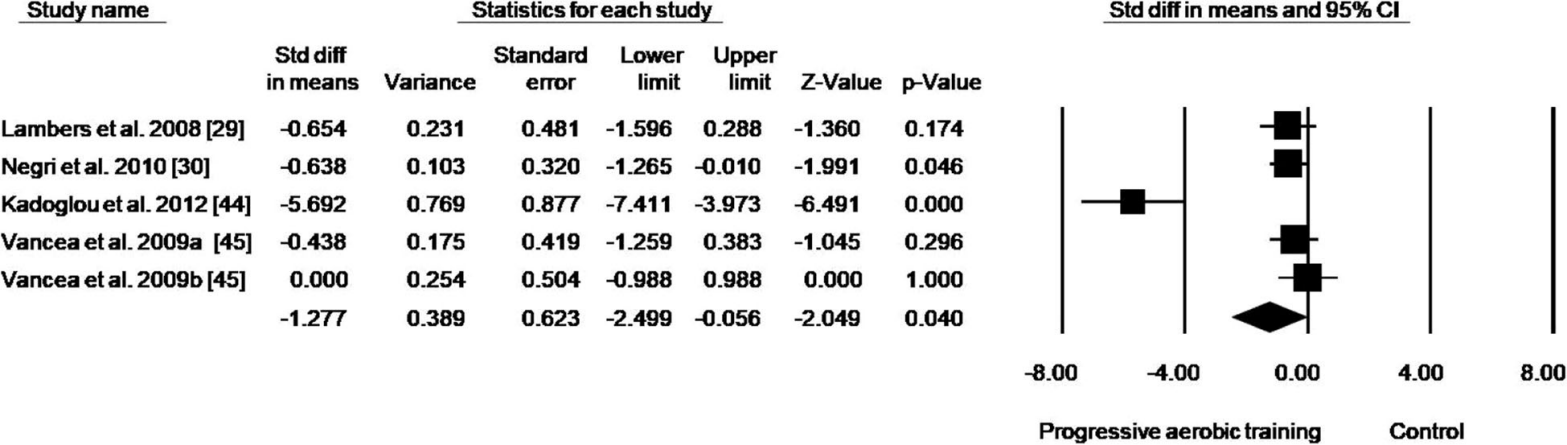
Standard mean differences in HbA1c observed with aerobic training progressing in intensity and control (no intervention). (Black square) Study-specific estimates; (black diamond) pooled estimates of random-effects meta-analyses. *Std diff* standardized difference, *CI* confidence interval. Letters (a and b) subscribed indicate different aerobic training protocols in a same study

**Fig. 5:**
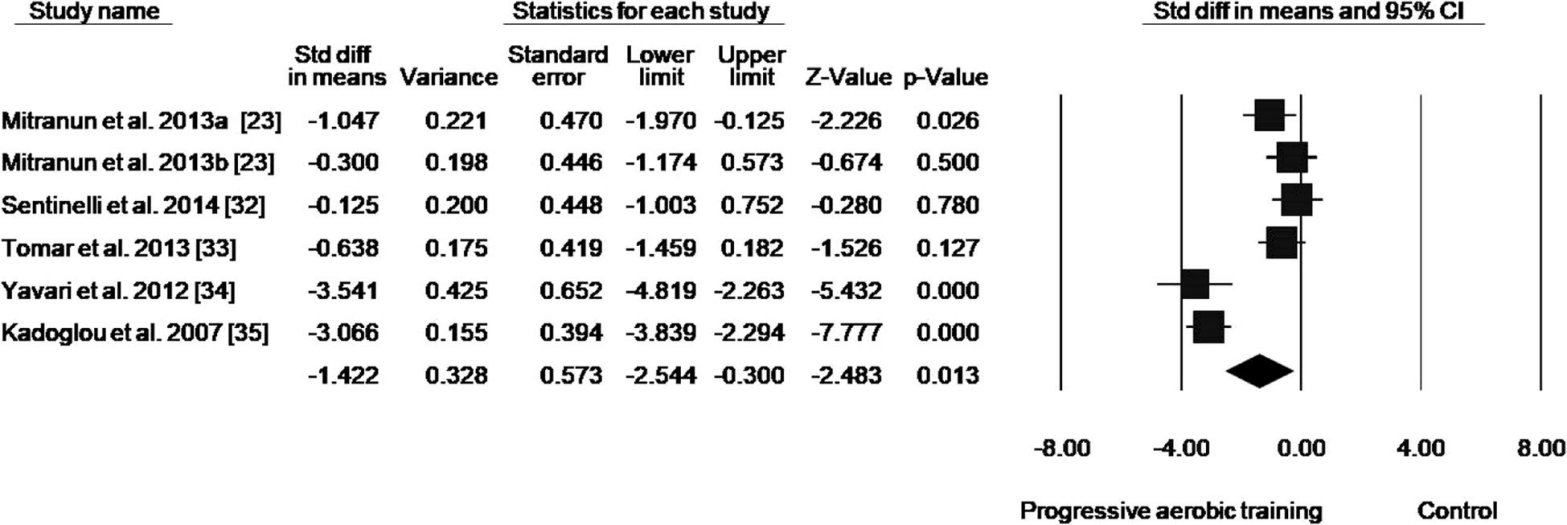
Standard mean differences in HbA1c observed with aerobic training progressing in both intensity and duration and with control (no intervention). (Black square) Study-specific estimates; (black diamond) pooled estimates of random-effects meta-analyses. *Std diff* standardized difference, *CI* confidence interval. Letters (a and b) subscribed indicate different aerobic training protocols in a same study

By analyzing only studies that progressed the training duration (volume), a reduction in HbA1c of 0.94% (effect size: − 1.967; 95% CI − 3.783, − 0.151; *p* = 0.034; *I*^2^: 85%) was detected (Fig. 3). Analysis of the studies that included progression only in intensity presented a reduction in HbA1c of 0.41% (effect size: − 1.277; 95% CI − 2.499, − 0.056; *p* = 0.040; *I*^2^: 88%) (Fig. 4). When analyzing studies that progressed both volume and intensity, a reduction of 1.27% (effect size: − 1.422; 95% CI − 2.544, − 0.300; *p* = 0.013; *I*^2^: 89%) in HbA1c was found (Fig. 5).

#### Meta-regression (Progressive)

According to the results of meta-regression analyses, mean age, percentage of women in the sample, diagnosis duration, baseline HbA1c values, weekly frequency, session duration, and weekly duration were not associated with the improvement in HbA1c caused by PAT (Table 5). However, BMI (β: − 0.737; 95% CI − 1.346, − 0.128; *p* = 0.017; *R*^2^: 0.20), number of glucose-lowering drug users (β: − 0.080; 95% CI − 0.113, − 0.046; *p* < 0.001; *R*^2^: 1.00), and follow-up duration (β: − 0.088; 95% CI − 0.147, − 0.029; *p* = 0.003; *R*^2^: 0.37) were inversely associated with the reduction in HbA1c caused by PAT.

**Table 5:** Meta-regression results

Moderator	Number of study estimates	*Β*	95% CI	*p* value	*R* ^2^
Progressive aerobic training					
Mean age	14	0.045	− 0.074; 0.166	0.457	0.00
Percentage of women in the sample	10	− 0.007	− 0.056; 0.041	0.765	0.00
Body mass index	13	− 0.737	− 1.346; − 0.128	*0*.*017*	0.20
Diagnosis duration	11	0.076	−0.069; 0.223	0.303	0.00
Baseline HbA1c	10	0.009	−1.508; 1.318	0.895	0.00
Glucose lowering drug users	5	− 0.080	− 0.113; − 0.046	< *0*.*001*	1.00
Follow-up duration	14	− 0.088	− 0.147; − 0.029	*0*.*003*	0.37
Weekly frequency	14	− 0.426	− 1.677; 0.825	0.504	0.00
Session duration	13	− 0.005	− 0.055; 0.043	0.820	0.00
Weekly duration	13	0.001	− 0.016; 0.017	0.960	0.00
Non-progressive aerobic training					
Mean age	12	0.001	− 0.001; 0.002	0.442	0.00
Percentage of women in the sample	10	0.003	− 0.013; 0.021	0.662	0.00
Body mass índex	12	0.096	− 0.039; 0.232	0.163	0.00
Diagnosis duration	10	− 0.015	− 0.449; 0.417	0.942	0.00
Baseline HbA1c	12	− 0.229	− 0.898; 0.440	0.502	0.06
Glucose lowering drug users	8	− 0.027	− 0.099; 0.045	0.462	0.00
Follow-up duration	13	0.005	− 0.046; 0.056	0.846	0.00
Session duration	13	− 0.026	− 0.058; 0.007	0.128	0.09
Weekly frequency	13	− 0.536	− 0.904; − 0.168	*0*.*004*	0.38
Weekly duration	13	− 0.005	− 0.010; − 0.001	*0*.*020*	0.27

Numbers in italic indicate statistical significance (*p* < 0.05)

### Effectiveness of Non-Progressive Aerobic Training

#### Meta-analysis Results (Non-Progressive)

Data concerning the HbA1c reduction associated with NPAT compared to control groups were available from 12 studies, with a total of 353 participants (NPAT groups = *n*: 274; control groups = *n*: 186) assessed (Fig. 2; Table 1). NPAT was associated with a reduction in HbA1c of 0.45% compared with no intervention (effect size: − 0.920; 95% CI − 1.329, − 0.512; *p* < 0.001; *I*^2^: 74%). The analysis of publication bias for this outcome showed no significant bias (*p* = 0.066).

#### Subgroup Analysis (Non-Progressive)

By analyzing trials in which only participants with comorbidities were included, NPAT was associated with a reduction of 0.33% (effect size: − 0.554; 95% CI − 1.048, − 0.061; *p* = 0.028; *I*^2^: 52%) in HbA1c, whereas a reduction of 0.79% (effect size: − 1.358; 95% CI − 2.556, − 0.159; *p* = 0.026; *I*^2^: 86%) was found in those that did not include participants with comorbidities. When analyzed according to the specific modality of AT, an HbA1c reduction of 0.43% (effect size: − 1.292; 95% CI − 1.856, − 0.727; *p* < 0.001; *I*^2^: 61%) for walking and/or running interventions was detected, whereas no HbA1c reduction was found when AT was performed on a cycle ergometer (effect size: − 0.156; 95% CI − 0.643, 0.330; *p* = 0.529; *I*^2^: 0%).

When divided according to the training status of the participants, studies that included untrained patients presented HbA1c reductions of 0.39% (effect size: − 0.956; 95% CI − 1.382, − 0.530; *p* < 0.001; *I*^2^: 58%), whereas studies lacking a clear description of the training status of the participants reports no HbA1c reduction (effect size: − 0.443; 95% CI − 1.646, 0.759; *p* = 0.470; *I*^2^: 84%).

#### Meta-regression (Non-Progressive)

According to the results of meta-regression analyses, mean age, percentage of women in the sample, BMI, diagnosis duration, baseline HbA1c, number of glucose-lowering drug users, and follow-up duration did not affect the improvement in HbA1c caused by NPAT (Table 5). However, weekly frequency (β: − 0.536; 95% CI − 0.904, − 0.168; *p* = 0.004; *R*^2^: 0.38) and weekly duration (β: − 0.005; 95% CI − 0.010, − 0.001; *p* = 0.020; *R*^2^: 0.27) of AT were inversely associated with the reduction in HbA1c caused by NPAT.

## Discussion

To the best of our knowledge, this is the first meta-analysis of the published studies investigating the progression of AT variables and its relationship with glycemic control in patients with type 2 diabetes. This meta-analysis included studies containing data of 825 participants. Our main results demonstrate that both training strategies (PAT and NPAT) are effective for inducing HbA1c reduction compared to no training. However, a greater magnitude of reduction was found when PAT was used.

The general finding (all groups of exercise vs. control groups) of this study (HbA1c − 0.65%) is in accordance with the average reductions in HbA1c indicated by the ADA recommendations (− 0.66%) [3] and with the findings of previous meta-analyses [8]. This further strengthens the evidence for the benefits of AT for glycemic control [47] and reinforces the effect of this type of training on other cardiovascular risk factors. Nevertheless, when analyzed separately, PAT presented a more substantial reduction (− 0.84%; ES: − 1.478; 95% CI − 2.197, − 0.759; *p* < 0.001; *I*^2^: 87%) in HbA1c than NPAT (− 0.45%; ES: − 0.920; 95% CI − 1.329, − 0.512; *p* < 0.001; *I*^2^: 74%). The magnitude of effect in the NPAT subgroup analysis was further reduced (− 0.37%) when only the studies with land-based aerobic training were analyzed, i.e., after exclusion from the analysis of the study by Nuttamonwarakul et al. [46], which evaluated aquatic training. This result has an important clinical implication because it does not only highlight the importance of the exercise dose (volume and intensity) but also highlights the relevance of the gradual increment in exercise dose throughout the course of the intervention. To date, previous studies [8, 13, 14] have provided evidence for the association of some training variables, such as weekly duration [8], weekly frequency [14], and intensity [13], with HbA1c reductions in patients with type 2 diabetes. With this in mind, our findings represent an advance in the literature, and provide evidence that even though training dose is important, the progression of the volume and intensity of exercise increases the extent of glycemic control promoted by AT.

Although the current guidelines [3, 4] suggest that physical activity should be progressed in terms of intensity, frequency, and/or duration, there is a lack of evidence regarding this process, mainly because progression is usually performed until the recommended exercise dosage is reached rather than as a constant practice by the professionals who prescribe exercise training to the diabetes population. The inconsistency of this issue becomes clear when analyzing the approaches of the studies included in this review. Exactly half of the studies did not involve progression of the volume and/or physiological intensity of training, although some did adjust the external load (i.e., velocity) while maintaining the same physiological intensity, representing a progression in absolute load but not in relative load. In contrast, the other half of studies progressed the physiological load throughout the intervention period, albeit without balancing the distribution of the load in different mesocycles. However, none of the studies was found to compare both training strategies (PAT vs. NPAT) with total work controlled, a requirement for increasing the understanding of the role of training progression and dosage (volume and intensity) in reducing HbA1c.

In the subgroup analysis of comorbidities, both training strategies (PAT and NPAT) were effective in patients with and without comorbidities. The effect was greater in patients without comorbidities and in those who participated in PAT. The benefits of PAT were evident because the reduction in HbA1c levels (− 0.79%; ES: − 3.061) found in patients with comorbidities, less responsive to training, who participated in PAT, was similar to the reduction found in patients without comorbidities, which are more responsive, to NPAT. The major responsivity to training in patients without comorbidities possibly may be due to the better training conditions/exercise tolerance of these patients.

By analyzing PAT modalities, a combination of different AT modalities provided HbA1c reductions (− 1.12%) of higher magnitude, despite the fact that a significant HbA1c reduction was also found when walking and/or running were performed alone (− 0.69%). A mixed strategy can favor motivation and adherence to training. However, this approach requires more effort to control the intensity of the different modalities performed. Conversely, when NPAT was performed, only walking and/or running were associated with HbA1c reduction (− 0.43%), whereas a significant HbA1c reduction was not found when using a cycle ergometer. This effect can be attributed to greater muscle mass involvement and consequently higher energy costs compared to cycle ergometer for the same relative intensity [48]. This finding corroborates previous findings of the effect of supervised walking on HbA1c reduction [9]. From the public health perspective, this can be considered as a positive result given that walking and running do not require equipment and are natural activities. However, because of the strong association between type 2 diabetes and obesity [49], sometimes referred to as “diabesity” [50], many patients may find it difficult to perform activities that require them to support their own body weight, such as walking or running, when attempting the recommended training durations and intensities.

Another subgroup analysis was performed to assess the training status of participants. This analysis only showed HbA1c reductions in untrained patients in both the PAT and NPAT groups. However, it is important to highlight that the majority of studies included (18 of 24, 75%) untrained patients, whereas in the other studies (six of 24, 25%), the training status of participants was not clearly described. It is possible that these studies included participants who were at least minimally trained, which could have decreased the degree of improvement. As the findings in these patients were not positive even when PAT was used, it is necessary to fill this gap in the literature. The importance of proposing training protocols for untrained patients is obvious in the context of a disease that is strongly associated with a sedentary lifestyle. Nonetheless, further progress is necessary to advance the structuring of training for active/trained patients. Thus, it will be possible to at least maintain the initial reductions but preferably continue reducing HbA1c to the desired values. With this, we believe that clinical trials of AT in patients with type 2 diabetes should include training periodization, which is considered to be a systematic variation in training specificity, intensity, and volume organized within cycles or periods of an overall program [51]. Overall, the impact of periodized training on health outcomes in untrained individuals is unclear, as this approach is typically used in sports training [52].

From the practical perspective, it is necessary to discuss the different forms of progression because in the context of training, it is possible to progress either the volume (frequency and duration) and/or intensity of training. By analyzing these different forms of progression, we found the highest HbA1c reduction (− 1.27%) when volume and intensity were incremented. When only volume was incremented, a substantial reduction was also found (− 0.94%), whereas increasing only intensity was associated with significant but less substantial HbA1c reduction (− 0.41%). These findings highlight the importance of quantitative progression, more precisely the session and week durations, as components to be incremented when the therapeutic goal is the glycemic control. Given that training duration does not completely represent training volume, it is important to highlight the possibility of investigating other alternatives of volume progression, such as increasing weekly frequency, a strategy that was not adopted in the included studies despite the fact that weekly frequency was the AT variable most associated with HbA1c reduction in a previous systematic review with meta-regression [14].

Meta-regression analyses were performed to clarify the possible moderators of the effects of AT on HbA1c. Interestingly, different moderators were found for PAT and NPAT. For PAT, BMI, number of glucose-lowering drug users, and follow-up duration were inversely associated with HbA1c reduction. The influence of BMI was in accordance with the subgroup analysis of comorbidities as overweight and obesity were the main comorbidities reported and these patients were less responsive to training. The greater HbA1c reduction found in participants with lower BMI may indicate the greater ease of these patients in adhering to and accomplishing the recommended dosages in the PAT models proposed, especially in the case of modalities that require the support of the patient’s own body weight. The inverse relationship between the number of glucose-lowering drug users and HbA1c reduction may be caused by the fact that these patients already have increased insulin sensitivity, among other therapeutic benefits of the pharmacological agents, whereas in non-users the effect of PAT appears to be more noticeable.

Another moderator that was inversely associated with HbA1c reduction was follow-up period, indicating that even with the progressive strategies used, long-term PAT interventions have not optimized the glycemic control. This result reinforces the need for studies of AT for type 2 diabetes management to better control and distribute the workloads in periodized models so that glycemic metabolism is impacted even after the patients have been trained and have adapted to AT.

In the meta-regression analyses performed with NPAT, weekly frequency and duration were inversely associated with HbA1c reduction. These findings were unexpected because of the known relationship between training volume and HbA1c reduction [8, 10, 14]. However, it is important to highlight that previous studies [8, 14] demonstrating the importance of volume variables did not evaluate progressive and non-progressive strategies separately, as in the present review, given that this is a novel discussion. A possible speculation about this finding is that when a non-progressive approach is adopted with a high volume, especially in untrained patients, the intensity is generally low. In this manner, volume is the only component of the dosage that can be considered as “high,” which in a fixed form does not seem to be associated with glycemic benefits.

As practical application, exercise professionals structuring AT for type 2 diabetes control should understand that the most important is the achievement of structured and supervised AT. However, HbA1c reductions of greater magnitudes occur when there is progression of training variables. Among possible progression strategies, our study showed more expressive HbA1c reductions when both duration and intensity are increased throughout the interventions, followed by progression in duration, with lower reductions with progression in intensity.

Finally, the high degree of heterogeneity of some comparisons and the poor methodological quality of some trials represent limitations of the present meta-analysis. Moreover, the vast majority of the studies included did not provide an adequate and detailed description of important methodological procedures, making it difficult to determine whether the risk of bias was high or low. Of the four criteria adopted for assessing the risk of bias, only two (random sequence generation and incomplete outcome data) were adopted in the majority of studies. The lack of some information about the training features and participants in some studies, such as the AT modality and training status of participants, is another limitation. Furthermore, the lack of registry of this study can be a limitation, making difficult for the reader to know if the methods used are in agreement with what was planned a priori. Nevertheless, the present meta-analysis contributes novelty to the literature in the area of exercise training and type 2 diabetes treatment, addressing a “new view” in which not only exercise dosage but also the progression of training variables (volume and intensity) over time should be considered when prescribing AT.

## Conclusions

Based on our results, AT (in general) is associated with a reduction in HbA1c in patients with type 2 diabetes. The effect of PAT was of greater magnitude than that of NPAT, especially when volume and intensity, or at least volume, was incremented during intervention. Therefore, the progression of AT variables should be considered in order to optimize glycemic control in patients with type 2 diabetes.

## Additional file

Additional file 1: Online Supplemental Material. (DOCX 19 kb)

## Abbreviations

AT: Aerobic training
HbA1c: Glycated hemoglobin
NPAT: Non-progressive aerobic training
PAT: Progressive aerobic training
RCTs: Randomized controlled trials
