# Supplementary material for: The Role of Aerobic Training Variables Progression on Glycemic Control of Patients with Type 2 Diabetes: a Systematic Review with Meta-analysis
Source: Sports Med Open. 2019 Jun 7;5:22. doi: 10.1186/s40798-019-0194-z (PMC6555839; doi:10.1186/s40798-019-0194-z)
Supplement: Supplementary file 1 — Online Supplemental Material. (DOCX 19 kb) [file 40798_2019_194_MOESM1_ESM.docx]

Online Supplemental Material

Supplemental Material 1.Search strategy used in PubMed.

| "Diabetes Mellitus, Type 2"[Mesh] OR "NIDDM"[title/abstract] OR "Maturity-Onset Diabetes"[title/abstract] OR "Diabetes Mellitus, Noninsulin-Dependent"[title/abstract] OR "Diabetes Mellitus, Adult-Onset"[title/abstract] OR "Diabetes Mellitus, Adult-Onset"[title/abstract] OR "Adult-Onset Diabetes Mellitus"[title/abstract] OR "Diabetes Mellitus, Adult Onset"[title/abstract] OR "Diabetes Mellitus, Ketosis-Resistant"[title/abstract] OR "Diabetes Mellitus, Ketosis Resistant"[title/abstract] OR "Ketosis-Resistant Diabetes Mellitus"[title/abstract] OR "Diabetes Mellitus, Maturity-Onset"[title/abstract] OR "Diabetes Mellitus, Maturity Onset"[title/abstract] OR "Diabetes Mellitus, Non Insulin Dependent"[title/abstract] OR "Diabetes Mellitus, Non-Insulin-Dependent"[title/abstract] OR "Non-Insulin-Dependent Diabetes Mellitus"[title/abstract] OR "Diabetes Mellitus, Noninsulin Dependent"[title/abstract] OR "Diabetes Mellitus, Noninsulin Dependent"[title/abstract] OR "Diabetes Mellitus, Slow-Onset"[title/abstract] OR "Diabetes Mellitus, Slow Onset"[title/abstract] OR "Slow-Onset Diabetes Mellitus"[title/abstract] OR "Diabetes Mellitus, Stable"[title/abstract] OR "Stable Diabetes Mellitus"[title/abstract] OR "Diabetes Mellitus, Type II"[title/abstract] OR "Maturity-Onset Diabetes Mellitus"[title/abstract] OR "Maturity Onset Diabetes Mellitus"[title/abstract] OR "MODY"[title/abstract] OR "Type 2 Diabetes Mellitus"[title/abstract] OR "Noninsulin-Dependent Diabetes Mellitus"[title/abstract] |
| --- |
| "Exercise"[Mesh] OR Exercises[title/abstract] OR "Exercise, Physical"[title/abstract] OR title/abstract OR "Exercises, Physical"[title/abstract] OR "Physical Exercise"[title/abstract] OR "Physical Exercises"[title/abstract] OR "Exercise, Isometric"[title/abstract] OR "Exercises, Isometric"[title/abstract] OR "Isometric Exercise"[title/abstract] OR "Isometric Exercises"[title/abstract] OR "Exercise, Aerobic"[title/abstract] OR "Aerobic Exercises"[title/abstract] OR "Exercises, Aerobic"[title/abstract] OR "Aerobic Exercises"[title/abstract] OR "Exercises, Aerobic"[title/abstract] OR "Aerobic Exercise"[title/abstract] |
| "randomized controlled trial[pt] OR controlled clinical trial[pt] OR randomized controlled trials[mh] OR random allocation[mh] OR double-blind method[mh] OR single-blind method[mh] OR clinical trial[pt] OR clinical trials[mh] OR ("clinical trial"[tw]) OR ((singl*[tw] OR doubl*[tw] OR trebl*[tw] OR tripl*[tw]) AND (mask*[tw] OR blind*[tw])) OR ("latin square"[tw]) OR placebos[mh] OR placebo*[tw] OR random*[tw] OR research design[mh:noexp] OR follow-up studies[mh] OR prospective studies[mh] OR cross-over studies[mh] OR control*[tw] OR prospectiv*[tw] OR volunteer*[tw]) NOT (animal[mh] NOT human[mh] |
